# Mid-Regional Proadrenomedullin as a New Biomarker of Kidney and Cardiovascular Diseases—Is It the Future?

**DOI:** 10.3390/jcm10030524

**Published:** 2021-02-02

**Authors:** Katarzyna Czajkowska, Edyta Zbroch, Angelika Bielach-Bazyluk, Katarzyna Mitrosz, Elzbieta Bujno, Katarzyna Kakareko, Alicja Rydzewska-Rosolowska, Tomasz Hryszko

**Affiliations:** 1 2nd Department of Nephrology and Hypertension with Dialysis Unit, Medical University of Bialystok, 15-276 Bialystok, Poland; b.angelika@wp.pl (A.B.-B.); katarzynamitrosz@gmail.com (K.M.); katarzyna.kakareko@umb.edu.pl (K.K.); alicja.rosolowska@umb.edu.pl (A.R.-R.); tomasz.hryszko@umb.edu.pl (T.H.); 2 Department of Internal Medicine and Hypertension, Medical University of Bialystok, 15-540 Bialystok, Poland; edyta.zbroch@umb.edu.pl (E.Z.); mlodawska.ela@gmail.com (E.B.)

**Keywords:** cardiovascular diseases, kidney diseases, mid-regional proadrenomedullin

## Abstract

The increasing prevalence of cardiovascular disease and concomitant chronic kidney disease among the aging populations is responsible for considerable growth of mortality. Additionally, frequent, prolonged hospitalizations and long-term treatment generates progressive decline in bodily functions as well as substantial public health and economic burden. Accessibility to easy, non-invasive prognostic markers able to detect patients at risk of cardiovascular events may improve effective therapy and mitigate disease progression. Moreover, an early diagnosis allows time for implementation of prophylactic and educational programs that may result in decreased morbidity, improved quality of life and reduced public health expenditure. One of the promising candidates for a novel cardiovascular biomarker is mid-regional proadrenomedullin, a derivative of adrenomedullin. Adrenomedullin is a peptide hormone known for its vasodilatory, antioxidant, antiapoptotic and antifibrotic effects. A remarkable advantage of mid-regional proadrenomedullin is its longer half-life which is a prerequisite for plasma measurements. These review aims to discuss the importance of mid-regional proadrenomedullin with reference to its usefulness as a biomarker of increased cardiovascular risk and kidney disease progression.

## 1. Introduction

The constantly growing world population and its parallel aging causes an increase in the number of chronically ill persons. Over time, most of them develop several comorbidities which further results in increased mortality. Cardiovascular disease (CVD) defined as a group of disorders affecting heart and blood vessels, is the most common non-communicable disease globally. According to the World Health Organization it is the leading cause of mortality with approximately 17 million deaths annually. Major cardiovascular risk factors include tobacco use, alcohol abuse, diet rich in saturated fat and refined carbohydrates and low physical activity. Unhealthy lifestyle also contributes to the development of hypertension (HT), diabetes mellitus, chronic kidney disease (CKD) and other organs injury. People with multiple CVD risk factors should be identified as early as possible to implement a strategy aimed at lowering risk factors, including lifestyle interventions and special treatment.

Chronic kidney disease is also classified as a lifestyle disease. It affects approximately 11–13% of the world population. CKD incidence increases with age and is more prevalent in women [1]. Due to asymptomatic onset early diagnosis remains challenging. Gradual decline in kidney function leads to the development of non-specific clinical symptoms, profound disturbances in mineral metabolism and accelerated cardiovascular disease. Undoubtedly, timely diagnosis is extremely important in the context of patients’ life expectancy, which can be extended by early implementation of lifestyle modification and treatment. CKD is often associated with hypertension (HT). A mutual relationship between high blood pressure and renal function contributes to development of volume-dependent hypertension in about 80% of CKD population, regardless of ethology [2]. Moreover, HT together with CKD are recognized as major cardiovascular risk factors and their frequent coexistence significantly increases cardiovascular morbidity and mortality [3]. Patients suffering from CVD frequently develop severe complications, including death.

Therefore, there is still an urgent need for developing novel biomarkers which allow accurate identification of high-risk patients.

The review aims to discuss the importance of mid-regional proadrenomedullin with reference to its usefulness as a biomarker of increased cardiovascular risk and renal disease progression.

## 2. Adrenomedullin vs. Mid-Regional Proadrenomedullin

**Figure 1:**
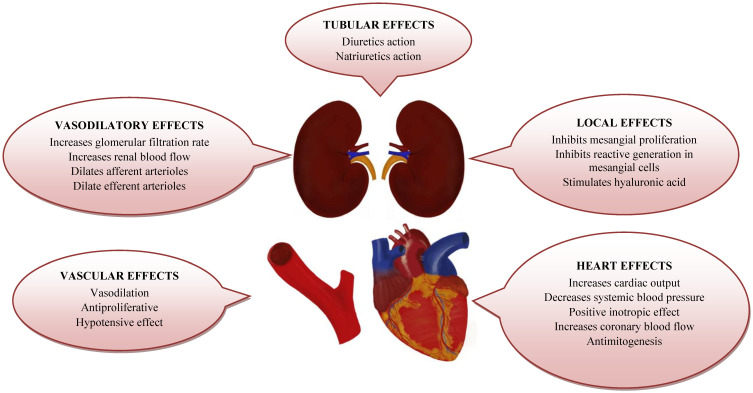
Effects of ADM on kidney and cardiovascular system.

One of candidates for a CKD and CVD biomarker is a peptide hormone, adrenomedullin (ADM). It was originally isolated from pheochromocytoma tissue in the end of the 20th century [4]. ADM shows natriuretic and vasodilatory properties, which result in a combined hypotensive effect (Figure 1). Growing amount of research confirms the expression of ADM receptors in many organs, including heart and vessels, muscles, endocrine glands, renal and nervous tissues. It was also detected at low concentration in serum and other body fluids [5]. The extensive presence of ADM suggests its involvement in functioning of various systems and organs. Unfortunately, its short half-life and in vitro instability make its direct measurement in serum impossible. Interestingly, an amino-acid sequence known as mid-regional proadrenomedullin (MR-ProADM) is released in equal concentration to ADM in the process of proteolytic cleavage [6]. It is a stable and inactive protein which can be easily detected. Hence, an enzyme-linked immunoassay was developed to assess its plasma concentration. So far, many studies attempted to establish the level of MR-ProADM in physiological conditions and diseases. Additionally, a remarkable piece of data provided evidence for the prognostic value of MR-ProADM in acute conditions as well as exacerbations of chronic diseases. The peptide was compared to others currently used biomarkers or added as a consecutive factor to various clinical assessment scales e.g., in sepsis [7] or in triage in the emergency department [8]. Majority of studies confirmed the significant relevance of MR-ProADM in the diagnosis, prognosis and estimation of mortality risk in hospitalized patients.

## 3. Impact of ADM on the Cardiovascular System

The influence of ADM on the cardiovascular system has been widely described many years ago. ADM through the vasodilation mechanism lowers blood pressure and increases the cardiac output [4]. What is interesting, it causes only slight stimulation of the sympathetic nervous system, resulting in tachycardia [9]. ADM is secreted by endothelial cells and binds to specific receptors causing vasodilation by increasing nitric oxide and cyclic guanosine monophosphate synthesis [10,11]. On the other hand, the role of ADM in myocardial contractility remains controversial. Previous studies conducted mainly on animal and human models obtained contradictory results (Table 1). In another one, ADM was isolated from cardiomyocytes and cardiac fibroblasts, which may suggest its involvement in the regulation of myocardial hypertrophy and remodelling secondary to hypertension [12,13].

The encouraging previous data on ADM biological effects have inspired researchers to undertake further studies on the role of its stable proteolytic cleavage MR-ProADM in several acute and chronic CVDs.

### 3.1. ADM and MR-ProADM in Heart Failure

Heart failure (HF) is a complex clinical syndrome occurring when cardiac output is insufficient. The study performed in 1999 found elevated plasma concentration of ADM in patients with congestive HF which correlated with the disease advancement [23]. ADM was found to be a negative prognostic marker of re-hospitalization, treatment effectiveness or disease exacerbations. Nishikimi et al. [24] also assessed ADM plasma level in patients with heart failure. They divided the study population into groups according to the New York Heart Association functional classes (NYHA) and the left ventricular ejection fraction determined by echocardiography. In the results, the authors found elevated ADM concentrations in the subsequent stages of heart failure and that the left ventricular ejection fraction inversely correlated with plasma ADM concentrations. More recent studies evaluated the value of MR-ProADM in comparison to other commonly used cardiovascular biomarkers (e.g., N-terminal-proBNP -NT-proBNP) regarding the risk of death [25] or re-hospitalization [26]. Molvin et al. [27] studied a group of 286 Swedish patients hospitalized due to chronic heart failure. During a follow-up period of approximately 17 months, 57 patients died and 90 required hospitalization again (because of HF exacerbation). All of them had an elevated level of MR-ProADM. Thus MR-ProADM predicted increased mortality risk and re-hospitalization after discharge.

The BACH Trial (Biomarkers in Acute Heart Failure) [28], conducted in patients with acute HF-related dyspnea referred to the emergency department, evaluated MR-ProADM plasma concentration comparing to other commonly performed cardiac biomarkers. It was a prospective international study involving 1641 patients, including 568 with HF. The prediction of 90-day survival of patients with heart failure was 73% for MR-ProADM and 62% for BNP (difference *p* < 0.001) and in adjusted multivariable Cox regression, MR-ProADM, but not BNP was an independent prognostic variable. MR-ProADM was also found a risk score predictor of long-term all-cause mortality in stable HF independent of BNP, echocardiographic abnormalities, clinical predictors of mortality and the Framingham score [29]. In turn, Billebeau et al. [30] compared patients with chronic heart failure and reduced left ventricular ejection fraction who underwent cardiac rehabilitation program to those treated without rehabilitation. MR-ProADM plasma concentration was assessed before and after rehabilitation (4–6 months). They found a decrease in MR-ProADM level in the group treated with cardiac rehabilitation together with an improvement of their general condition. Another study investigated the value of MR-ProADM as a biomarker in screening tests for left ventricular hypertrophy (LVH) in patients with hypertension [31]. LVH is a compensatory mechanism to left ventricular pressure overload that develops as one of many possible HT complications. MR-ProADM plasma level was substantially higher in patients with HT and LVH than those without LVH based on echocardiography. Additionally, the authors suggested practical applicability of the MR-ProADM plasma level measurement as a rule-out test for LVH in hypertensive patients. Due to low test specificity, it was recommended for patients who screened positive to be further evaluated with echocardiography.

#### Cardiorenal Syndrome

Patients with decompensated heart and significant renal failure often require urgent hospitalization. Usually, in this population, the diagnosis of a cardiorenal syndrome requires rapid and intensive management. So far, the search for prognostic biomarkers in this group was mostly unsuccessful, as the cause of exacerbation may be renal or cardiological. Regarding existing evidence on acute heart failure, the studies revealed association between increased ADM or MR-ProADM levels and prediction of treatment failure.

The study conducted on 82 patients with diabetes, researchers assessed the ADM levels in a subclinical cardiorenal syndrome defined as mild-to-moderate decline of renal function and LV relaxation impairment [32]. ADM concentration and GFR rate were compared to changes in left ventricular end-diastolic pressure. The obtained results confirmed that ADM modulates the relationship between the heart and kidneys in early subclinical cardiorenal syndrome. The increased ADM level was associated with a worse prognosis. Additionally, ADM was found to prevent the development of diastolic heart failure and the progression of renal dysfunction at the initial stage of cardiorenal syndrome. Due to lack of data future research should continue to explore the relevance of MR-ProADM in the cardiorenal syndrome, particularly as a prognostic or disease biomarker.

### 3.2. ADM and MR-ProADM in Myocardial Infarction

In 1998, Yoshitomi et al. [33] evaluated plasma ADM in myocardial infarction. They measured ADM level twice—one day after myocardial infraction and then after four weeks. Plasma ADM level increased in the early phase of acute myocardial infarction proportionally to its clinical severity and it was further elevated in patients with congestive heart failure.

Furthermore, in the LAMP Study (Leicester Acute Myocardial Infarction Peptide) [34] a significant increase in plasma MR-ProADM after myocardial infarction correlated with poor cardiac outcomes. Additionally, Arrigo et al. [35] described a correlation between MR-ProADM level and acute myocardial infarction severity with the highest concentration of the biomarker found in patients with cardiogenic shock. According to the study, elevated MR-ProADM level at the admission was associated with higher risk for acute HF during hospitalization. As described by Walter et al. [36], MR-ProADM and NT-proBNP were valuable predictive markers for mortality risk in acute myocardial infarction as well as in other long-term adverse clinical outcomes including recurrent acute myocardial infarction, congestive heart failure, cardiopulmonary resuscitation and cardiogenic shock or syncope.

ADM performed protective through several mechanisms: vasodilatation of the coronary vessels and increased myocardial blood flow, altering maladaptive cardiac remodeling by virtue of antioxidant, antiapoptotic and antifibrotic effects.

### 3.3. Significance of ADM and MR-ProADM in Hypertension

Plasma ADM levels are increased in patients with high blood pressure, particularly in those with uncontrolled hypertension and consequently left ventricular hypertrophy. Whether a novel hypotensive drug could be developed on the basis of the vasodilatory effect of ADM is an important question to be addressed. So far, the usage of ADM as a therapeutic agent decreasing blood pressure was assessed in spontaneously hypertensive rats [37].

One of the first publications aimed ADM concentration in patients with hypertension and kidney failure was a research of Ishimitsu et al. [38]. The study included persons with HT, stable chronic kidney disease and a control group. The results revealed an increase of plasma ADM concentration in whole studied population then in healthy individuals and what is even more interesting, it was higher in subjects with kidney failure comparing to those with hypertension. In turn, ADM level was significantly elevated in patients with HT and organ damage.

On the other hand, Kato et al. [39] assessed plasma ADM concentration in patients with primary and malignant hypertension and found it increased in malignant HT with a significant decline after effective hypotensive treatment. Then, in 2006 Kato et al. [40] analysed ADM level in a study group including subjects without overt cardiovascular or kidney disease. The overall aim was to investigate whether a rise of ADM level predicted the development of HT. The participants were divided into two groups according to ADM cut-off level and then followed up for three years. Interestingly, the results demonstrated two points. First—plasma ADM level was elevated before the development of hypertension. Second—ADM level was associated with age and increased BMI in normotensive persons. Hu et al. [41] examined changes in plasma ADM concentration in patients with primary hypertension (defined as a systolic blood pressure equal to or greater than 140 mmHg or a diastolic pressure equal to or greater than 90 mmHg, or both), without comorbidities compared to patients with borderline and normal blood pressure. Plasma ADM concentration was measured before the implementation of antihypertensive treatment and after 4 weeks of effective therapy. It was significantly decreased after four weeks of effective antihypertensive therapy. Furthermore, ADM concentrations strongly correlated with serum creatinine levels which may have resulted from kidney damage caused by hypertension.

The renin-angiotensin-aldosterone system (RAS) plays a pivotal role in the development of hypertension. Charles et al. [42] in their interesting research described the effect of ADM infusion on RAS in two models—animal and human. In human model healthy individuals, then patients with heart failure, hypertension or chronic kidney disease were included. The results revealed ADM acting as a functional antagonist of angiotensin II and then as an inhibitor of aldosterone secretion what caused BP lowering. Another study designed to measure ADM levels in three groups of hypertensive patients: with low, medium or high renin levels, revealed increased plasma ADM concentration only in the high renin activity group [43]. Going further, Italian researchers also assessed ADM plasma concentration in patients with hypertension [44]. The studied population was divided into patients with malignant HT, renovascular HT (RAS is significantly active in both of those groups) comparing to subjects with primary HT and normotensive group. The results again confirmed an increase of ADM concentration in proportion to the severity of HT. Much higher values were observed in the malignant and renovascular HT, which indicated that the activation of RAS might contribute to the ADM level elevation. The large KORA F4 (Cooperative Health Research in the Region of Augsburg, southern Germany) study, which enrolled 1261 participants, assessed the RAS relationship with MR-ProANP and MR-ProADM in patients with type 2 diabetes or pre-diabetes [45]. Surprisingly, no significant correlation of studied peptides with RAS in patients with diabetes or pre-diabetes was revealed. The results are opposite to those found in non-diabetic persons therefore require further confirmation.

### 3.4. Possible Value of ADM or MR-ProADM in Other CVD

Overtime, extensive literature has appeared on the value of MR-ProADM in less prevalent conditions from the spectrum of cardiovascular diseases. In 2008, study on MR-ProADM usefulness for the assessment of left ventricular ejection fraction in patients with coronary artery disease found that it may be used as a marker of left ventricular dysfunction in outpatient care before further imaging diagnostics [46]. This conclusion was determined on the basis of plasma MR-ProADM measurement and echocardiography performed at the same time and about 687 days followed up. It is plausible that in the future, a simple outpatient plasma MR-ProADM concentration measurement may sometimes replace echocardiography, which requires qualified personnel and an appropriate ultrasound scanner.

Going further, Elmaset al. [47] assessed the value of several cardiac markers including MR-ProADM in prediction of atrial fibrillation (AF) relapse after pulmonary vein isolation. The results suggest that MR-ProADM and NT-proBNP could act as predictors of AF recurrence and may help to establish a therapeutic approach which is important to optimize treatment.

A Swedish study examined plasma MR-ProADM level as a biomarker of an abdominal aortic aneurysm (AAA) [48]. The prospective study (follow-up of about 12 years) of a population of 5551 patients demonstrated elevated concentration of plasma MR-ProADM associated with the AAA development.

Another research revealed that plasma MR-ProADM concentration analysis in patients after trans catheter aortic valve implantation (before intervention, 30-days and 1-year after intervention) may improve patient’s monitoring within one year of observation and may help to identify patients at higher risk of cardiovascular events and all-cause mortality [49].

The use of ADM as a drug has been under research for several years. In 2009, Japanese authors performed a study on a small group of people in which the effectiveness of ADM as a drug was assessed. They observed a beneficial hemodynamic and hormonal effect after intravenous administration of ADM together with human natriuretic peptide in acute decompensated heart failure [50]. Similar findings were obtained in a study on patients with congestive heart failure. Infusion of ADM resulted in heart and kidney function improvement [51]. Research evaluating the effectiveness of ADM as a drug is still ongoing and full evaluation of its effectiveness is not currently possible.

In conclusion, the literature pertaining to MR-ProADM in CVD strongly suggests its supportive role in patient classification regarding the risk of possible complications.

## 4. Significance of ADM in Kidney Diseases

In the kidney ADM is produced in glomeruli, distal tubules, medullary collecting duct and mesangial cells (Table 2) [52]. In 2007, Nishikimi et al. [53] summarized the current state of knowledge on the role of ADM in renal physiology and pathophysiology. Plasma ADM levels increased in patients with kidney impairment and correlated with severity of the disease. The analysis also showed ADM involvement in hemodynamic regulation, glomerular filtration rate (GFR) and water and sodium homeostasis. In particular, ADM increased renal blood flow, GFR, natriuresis and inhibited mesangial proliferation. Several malignant hypertension rodent models have described improvement in glomerular sclerosis, fibrosis and arteriosclerosis after therapy with ADM infusion.

### 4.1. ADM and MR-ProADM in Chronic Kidney Disease

Since the renoprotective properties of ADM were established, a compensatory role of increased ADM concentration in CKD was suggested. Elevated peptide concentration may be a significant predictor of kidney failure progression. Moreover, it may be protective against ischemia-reperfusion injury.

In the MMKD (Mild-to-Moderate Kidney Disease) Study [61], MR-ProADM level was assessed in relation to stages of CKD advancement in primary CKD population without diabetes. The 7-year observation revealed continuous increase in MR-ProADM plasma level across all CKD stages, indicating an association with disease severity. In addition, elevated plasma levels of MR-ProANP and MR-ProADM at baseline were strong predictors of renal endpoints. Ishimitsu et al. [38] also described the relationship between ADM level increase and body fluid volume change in hypertension and kidney failure. The increase of plasma ADM level was more prominent in kidney failure than in hypertension. Additionally, in patients with CKD and hypertension an increase in ADM was more pronounced than in patients with vascular complications.

### 4.2. Significance of MR-ProADM and ADM in Acute Kidney Injury (AKI)

In 1999 Japanese researchers using animal models (dog kidney cells, rat vascular smooth muscle cells and rat mesangial cells) determined the contribution of ADM in AKI due to cell hypoxia [62]. The study was conducted under normoxic and hypoxic conditions. The results revealed that hypoxia up-regulated ADM gene expression in kidney and then increased its concentration. Nishimatsu et al. [63] analysed the ADM involvement in kidney injury due to ischemia followed by reperfusion in mice. They found ADM deficiency aggravated and overexpression limited histological lesions and functional impairment. The above results indicated a protective effect of ADM on AKI. Our previous study evaluated utility of MR-ProADM in AKI prediction in critically ill patients [64]. The MR-ProADM levels did not correlate with AKI incidents, but discriminated patients with high odds of death. The latest study of Liu et al. [65] performed in a group of critically ill sepsis patients suggested that ADM may become a biomarker for rapid assessment of the presence and severity of AKI at an early stage.

Due to the small amount of studies and the diverse study groups, it is difficult to determine the influence of MR-ProADM and ADM on AKI. This issue requires further research.

### 4.3. ADM and MR-ProADM in Glomerulonephritis

Plasma ADM concentration was also assessed in patients with glomerulonephritis. A research by Kuo et al. [66] aimed to measure synthesis of glomerular ADM in patients with IgA nephropathy. The authors reported that glomerular production of ADM was decreased in patients with IgA nephropathy and the decrease occurred earlier than the onset of renal failure. The decline in glomerular ADM production was responsible for lower urine ADM levels. In contrast, production in glomeruli was not related to plasma ADM levels. In addition, the lack of glomerular ADM may be associated with the proliferation of glomerular mesangial cells, which is observed in patients with IgA nephropathy. Another study of patients with chronic glomerulonephritis (minimal changes nephtotic syndrome, focal segmental glomerulosclerosis, membranous nephropathy) assessed plasma and urinary levels of ADM [67]. In the first step, the authors found plasma ADM levels were higher and urinary levels were lower in patients with glomerulonephritis comparing to the healthy control group. In the next step, study population was divided into two groups depending on daily urine protein excretion. In result plasma ADM concentration correlated positively and urinary level correlated negatively with the degree of proteinuria. This finding suggests that plasma and urine ADM concentrations may reflect disease activity or glomerular damage.

In turn, Mak et.al. [68] examined plasma ADM level in patients with systematic lupus erythematosus divided into groups according to lupus nephritis occurrence based on kidney biopsy specimens. The plasma ADM concentration was elevated in lupus nephritis and correlated with systemic lupus erythematosus disease activity.

The paucity of data on ADM level in glomerulonephritis prevents from a thorough review of these nephropathies.

### 4.4. MR-ProADM in End-Stage Kidney Disease (ESKD)

Many experiments regarding MR-ProADM plasma concentration were also carried out in patients on renal replacement therapy. Haemodialysis patients were the most widely studied population. The plasma level of MR-ProADM in that group was significantly elevated due to both reduced clearance and accumulation. MR-ProADM was found to be a strong predictor of all-cause and cardiovascular mortality within a 3.8-year follow-up in haemodialysis patients [69]. The same findings came from the study published three years earlier [70].

Another study of 70 haemodialysis patients with cardiac dysfunction demonstrated a significant decrease in MR-ProADM plasma concentration during haemodialysis [71]. MR-ProADM level also reflected left ventricular dysfunction (systolic and diastolic) and excessive blood volume in that population. Additionally, patients with higher MR-ProADM level had a higher mortality comparing to those with low concentration. It was another proof of MR-ProADM validity for assessing the severity of LV dysfunction and excessive blood volume.

Similar findings regarding the plasma level of ADM in haemodialysis patients were observed by Toepfer et al. [72]. It was elevated compared to the control group. Interestingly, patients with coexisting HF or HT had a significantly higher level of ADM than patients with ESKD alone. Additionally, ADM level before haemodialysis and 14–20 h after correlated with blood pressure.

Dialysis patients represent a population with the worst prognosis. CVD and excess fluid are the main causes of mortality. That is why, proper screen testing may result in earlier treatment implementation and therefore lead to improved survival in dialysis population.

### 4.5. MR-ProADM after Renal Transplantation

A study demonstrating changes in MR-ProADM level in patients who underwent kidney transplantation showed relationship between MR-ProADM concentration and CKD progression. Suzuki et al. [73] on a group of 11 renal recipients showed a significant decline in plasma concentration of MR-ProADM in the following weeks after kidney transplantation. Improvement in kidney function after renal transplantation resulted in decreased MR-ProADM level and showed that it might be a new promising biomarker. The same researchers in the next study on kidney transplant recipients found MR-proADM concentration were an independent factor associated with the intensity of hypotensive treatment [74] and indicated resistance to antihypertensive therapy. The conclusions drawn from this study emphasized the correlation between treatment intensity score of antihypertensive (daily dose taken by the patient divided by the maximum recommended daily dose for each of the taken antihypertensive drugs) and MR-proADM plasma level.

## 5. NT-ProBNP vs. ADM and MR-ProADM

The first studies on BNP and NT-proBNP dates back to 1988 [75]. NT-proBNP, similarly to MR-ProADM, is a biologically inactive peptide released from the heart in response to changes in ventricular filling pressures. It possesses a broad spectrum of action, including hypotensive, natriuretic, and diuretic properties, which together lead to its common use in everyday medical practice as cardiovascular marker. The primary application of NT-proBNP measurements is diagnosis, treatment evaluation and prognosis of heart failure. The obtained results should be interpreted with caution as several factors influence its plasma level. Among the others, age, sex, body weight, kidney function, the hydration status, type of assay applied, and genetic conditions affects the level [76,77,78]. It should be stressed NT-proBNP is not recommended for the diagnosis of HF in patients with kidney failure or on dialysis [79,80]. 

To date, considerable research efforts have been devoted to the determination of NT-proBNP function and relevance in patients with HF [81,82,83,84,85] and other cardiovascular diseases [86,87,88]. Strong correlations and easy assessment in plasma caused inclusion of NT-proBNP to routinely be used in cardiac testing panels. Over the past years, the relationship between ADM and BNP (or MR-proADM and NT-proBNP) have been analysed concomitantly in various diseases [89,90,91,92]. The results were often contradictory probably due to discrepancies in studied population or methodology so that topic still needs the further evaluation.

Despite well demonstrated relevance in cardiovascular and kidney disease, MR-proADM requires further estimation in large-scale trials to be introduced in everyday clinical practice.

## 6. Conclusions

The aging of the world population and better control of chronic diseases resulted in an increased number of people with CVD and CKD. Comorbidity increases cardiovascular risk, so new biomarkers are needed to predict disease progression and prognosis. The coexistence of heart failure or arterial hypertension in patients with kidney failure increases the mortality risk. The possibility of using renal replacement therapy also extends life expectancy of patients with ERSD. However, complications from CVD can reduce their quality of life and are the main cause of morbidity and mortality. Extensive research supports the significance of MR-proADM as a prognostic biomarker in both CVD and kidney diseases. Previous studies assessed the plasma level of MR-proADM in emergencies and chronic diseases. Consistently a predictive role was demonstrated However, further research is required to confirm the above. Additionally, the ongoing studies regarding the use of ADM as a promising therapeutic agent shed hope of both CVD and CKD patients.

## Figures and Tables

**Table 1:** Studies evaluating effects of ADM on cardiovascular system.

Author	Population	Organ	Effect
Lippton (1994) [14]	rat	pulmonary vascular	decreased lobar arterial pressure highest doses mildly decreased systemic arterial pressure
Lainchbury (1997) [15]	human	vascular	reduced mean arterial pressure reduced systolic arterial pressure reduced diastolic arterial pressure
Parkes (1997) [16]	sheep	vascular, heart	vasodilation increased cardiac contractility and cardiac output hypotensive actions
Ikenouchi (1997) [17]	rabbit	cardiomyocytes	negative inotropic effect
Yoshimoto (1998) [18]	rat, pig	rat aorta, pig coronary artery	vasodilation
Sait (1998) [19]	rat	brain ventricular	induced hypertension, tachycardia central regulation of the cardiovascular system
Szokodi (1998) [20]	rat	heart	enhanced cardiac contractility
Ihara (2000) [21]	rat	papillary muscles	positive inotropic effects increased intracellular contents of cAMP
Saetrum Opgaard (2000) [22]	human	cardiomyocytes	no positive or negative inotropic actions

**Table 2:** Studies evaluating the effects of ADM on kidney.

Author	Population	Organ	Effect
Ebara (1994) [54]	dog	kidney	vasodilatation increased urine flow increased urinary excretion of sodium and potassium with no effect onrenin activity
Jougasaki (1995) [55]	dog	kidney	increased GFR decrease in distal tubular sodium reabsorption, natriuretic effect, diuretic effect
Hirata (1995) [10]	rat	kidney	increased diameters of both afferent and efferent arterioles reduction in BP vasodilatation
Vari (1996) [56]	rat	kidney	minimal hypotensive effect increased renal plasma flow natiuretic effect
Majid (1996) [57]	dog	kidney	decreased renal vascular resistance increased renal blood flow no change ofGFR regulation ofwater and electrolyte excretion
Owada (1997) [58]	rat	nephron, kidney	increased GFR diureticeffect
Jensen (1997) [59]	rat	Juxtaglomerulargranularcells, kidney	stimulatory effect on renin secretion
Leclerc (2000) [60]	human	proximal and distal tubule, kidney	natriuretic effect

## Data Availability

No new data were created or analyzed in this study. Data sharing is not applicable to this article.
